# The role of TLR4/MyD88 signaling in systemic inflammation in an Alzheimer mouse model

**DOI:** 10.1002/alz.094976

**Published:** 2025-01-09

**Authors:** Junling Yang, Ken‐ichiro Fukuchi

**Affiliations:** ^1^ University of Illinois College of Medicine at Peoria, Peoria, IL USA

## Abstract

**Background:**

Many lines of evidence support that systemic inflammation promotes Alzheimer’s disease (AD) progression. Amyloid plaques are closely accompanied with neuroinflammation characterized by activated microglia and reactive astrocytes. Aging is the largest known risk factor for AD and is characterized by chronic, systemic, low‐grade inflammation (inflamm‐aging). However, the precise molecular mechanisms by which chronic, systemic inflammation contributes to development of AD remain to be elucidated. In order to investigate the role of TLR/MyD88 signaling in chronic, systemic, low‐grade inflammation associated with development of AD, an AD mouse model (TgAPP/PS1) with MyD88+/+ (wild‐type) or MyD88± (MyD88 heterozygous: one allele is deleted) was treated with low dose lipopolysaccharide (LPS) for 3 months.

**Method:**

We treated 6‐month‐old female MyD88+/+ TgAPP/PS1 and MyD88± TgAPP/PS1 mice with LPS (0.5 mg/Kg/weekly intraperitoneal injection) or PBS for 3 months and, then, performed a battery of behavioral tests including open‐field, T‐maze, elevated plus‐maze, and Morris water maze (n = 6‐7/group).

**Result:**

In the open‐field, LPS‐treated MyD88± TgAPP/PS1 mice spent more time in the periphery (P<0.05) and tended to spend less time in the center of the open‐field, suggesting increased anxiety. In the acquisition phase of Morris water maze, there were significant day effects for path lengths (F (4, 84) = 24.66, P< 0.001) and escape latencies (F (4, 84) = 23.39, P < 0.001) caused by a reduction in both values over days in all 4 groups. There were no intergroup differences in the path lengths and the latencies. In the probe trial, the percentages of time spent in the correct quadrant for LPS‐treated MyD88+/+ TgAPP/PS1 mice were significantly greater than any other groups (F (3, 21) = 5.46, P<0.01). There were no intergroup differences in the visible platform subtask.

**Conclusion:**

Surprisingly, chronic and systemic LPS treatment improved memory only in MyD88+/+ TgAPP/PS1 mice but not in MyD88± TgAPP/PS1 mice. We are currently investigating pathological and biochemical changes in these experimental animals.

